# Effects of the Sport Health-Related Fitness Program on Muscular Endurance, Flexibility, Cardiovascular Endurance, and Muscular Strength and Power in Korean College Students

**Published:** 2019-03

**Authors:** Eun-Hye KIM, Wi-Young SO

**Affiliations:** 1. Department of Physical Education, Korea University, Seoul, Korea; 2. Sports and Health Care Major, Korea National University of Transportation, Chungju-si, Korea

## Dear Editor-in-Chief

The percentage of Korean college students who participate in some form of physical activity three times a week is only 28.4%. Adults over the age of 20 yr are the only group that participates in regular exercise on a less frequent basis (22.8%) than Korean college students (1). Due to time constraints related to their studies and job searches, the amount of time that these students have for physical activity is estimated to be about 18 min per week (2). In line with global increases in obesity rates among people in their 20s (3), the obesity rate among Korean college students rose dramatically from 15.2% in 1998 to 23.9% in 2014, which is a significant increase compared to other age groups (4). Decreased participation in physical activity results in deteriorating physical fitness and an increased risk of obesity and other metabolic syndrome risk factors (5–6). Therefore, to improve college students’ physical fitness levels, health-related fitness programs are required.

The purpose of the health-related fitness program is not only to improve physical fitness during the college years but also to develop lifelong exercise and physical fitness habits. College students, however, often view exercise as strenuous and tiresome because of the perception that repetition and overload training are required. Therefore, the health-related fitness program has adopted the New Sports method to circumvent these negative impressions and promote positive attitudes towards exercise and physical fitness. This study aimed to verify the effectiveness of the ‘Sport Health-Related Fitness’ programs, such as New Sports, in improving college students’ physical fitness.

Overall, 1614 college freshmen (941 men and 673 women) who took the ‘Sport Health-Related Fitness’ program at Daejeon University, Daejeon-si, Korea from 2013 to 2015 participated in the study. The participants underwent health-related fitness measurements before and after participating in the course, which required 100 min of physical fitness activity per week for 14 wk. The ‘Sport Health-Related Fitness’ Program is described in Table 1.

**Table 1:** ‘Sport Health-Related Fitness’ program description

***Week***	***Level***	***Time (minutes)***	***Content***
	Warm-up	10	400m walking 1 set
		10	Light stretching, 1 set
3–8	Main exercise	30	Squat, lunge, side flank, knee push-up, sit-ups burpee
		10	Break time
		40	Dodge ball, flying- disc, handler, kinball, floorball, teeball
9–14		30	Run: 400m, step-ladder, mini hurdles, rubber cone
		10	Break time
		40	Squat, lunge, side flank, knee push-up, sit-ups burpee
15		30	Run: cycle ergometer, treadmill
		10	Break time
		40	Back extension, bench-press, pull down, leg extension, leg curl, leg press, pec-deck flyer, arm curl, hack squat
	Cool-down	10	Light stretching, 1 set

Informed consent was obtained from each student prior to their participation in the study. Based on the American College of Sports Medicine (7) and Ministry of Culture, Sports and Tourism of Korea (8), the health-related fitness test consisted of factors that evaluated muscular endurance, flexibility, cardiovascular endurance, and muscular strength and power.

The data were analyzed with SPSS Ver. 18.0 (SPSS, Chicago, IL, USA), used to calculate the means and standard deviations. Paired t-tests were used to analyze the effectiveness of the ‘Sport Health-Related Fitness’ program. Statistical significance was set at *P*<0.05. After the 14-week ‘Sport Health-Related Fitness’ program, a significant improvement was seen in muscular endurance, flexibility, cardiovascular endurance, and 50 m muscular strength and power in both men and women (*P*<0.001) (Table 2).

**Table 2:** Changes in muscular endurance, flexibility, cardiorespiratory endurance, and muscular strength and power after 14 wk of the ‘Sport Health-Related Fitness’ program

***Variables***	***Group***	***Pre***	***Post***	***t***	***P***
Muscular endurance: Sit-ups(reps/min)	Total	34.06±13.87	38.03±13.17	−20.842	<0.001***
	Men	40.45±11.32	44.39±10.50	−15.933	<0.001***
	Women	25.13±12.04	29.14±11.24	−13.429	<0.001***
Flexibility: Sit and reach(cm)	Total	12.08±9.22	14.39±8.33	−19.234	<0.001***
	Men	10.01±9.14	12.12±8.23	−12.931	<0.001***
	Women	14.99±8.54	17.63±7.27	−14.974	<0.001***
Cardiorespiratory endurance: PACER(reps)	Total	40.36±17.85	45.34±17.58	−19.825	<0.001***
	Men	50.76±15.47	55.83±14.43	−13.290	<0.001***
	Women	25.83±8.18	30.66±8.96	−17.419	<0.001***
Muscular strength and Power: 50 m dash(sec)	Total	8.74±1.50	8.39±1.27	16.438	<0.001***
	Men	7.84±1.05	7.55±0.70	10.178	<0.001***
	Women	9.99±1.09	9.56±0.91	13.653	<0.001***

Total=1,614; men=941; women=673

Data are presented as means±standard deviations

*** *P*<0.001; tested using paired *t*-tests

The ‘Sport Health-Related Fitness’ program improved the health-related fitness of college students in their 20s. Thus, development and distribution of these types of exercise programs are needed to enhance the health and wellness of students during their college years and beyond.
